# A95 APPROACH AND OUTCOMES OF PATIENTS WITH NON-VARICEAL UPPER GASTROINTESTINAL BLEEDING: ANALYSIS OF 50 CASES IN A TERTIARY CARE CENTRE

**DOI:** 10.1093/jcag/gwae059.095

**Published:** 2025-02-10

**Authors:** K A Labib, S Zepeda-Gomez, a aldaihani, M Fazal, A Hashmi

**Affiliations:** Gastroenterology, University of Alberta, Edmonton, AB, Canada; Gastroenterology, University of Alberta, Edmonton, AB, Canada; Gastroenterology, University of Alberta, Edmonton, AB, Canada; Gastroenterology, University of Alberta, Edmonton, AB, Canada; Gastroenterology, University of Alberta, Edmonton, AB, Canada

## Abstract

**Background:**

Non-variceal upper gastrointestinal bleeding (NVUGIB) is a significant cause of morbidity and mortality worldwide. NVUGIB refers to bleeding occurring above the ligament of Treitz, excluding variceal causes. Despite improvements in management, the incidence and mortality of NVUGIB remain high. Key challenges include determining optimal treatment strategies and predicting patient outcomes.

**Aims:**

To analyze clinical presentation, management strategies, endoscopic findings, treatment and outcomes of NVUGIB patients.

**Methods:**

A retrospective review was conducted at the University of Alberta Hospital on 50 patients diagnosed with NVUGIB who underwent endoscopy within 48 hours of presentation. We analyzed clinical presentation, management strategies, endoscopic findings, treatment and outcomes to assess their impact on patient care.

**Results:**

Of the 50 patients, 54% were male, and melena was the most frequent presenting symptom (56%). Sixty-two percent of patients received intravenous proton pump inhibitors (IV PPI) during initial management, while 42% were on NSAIDs without concurrent PPI therapy. Peptic ulcer disease was the leading cause of bleeding (90%), followed by vascular lesions (6%) and Mallory-Weiss tears (4%). Among ulcers, 66.7% were classified as Forrest III. Nine patients (18%) underwent urgent endoscopy within 6-12 hours, while 30% had early endoscopy (12-24 hours), and 52% had late endoscopy (after 24 hours). High-risk lesions (Forrest Ia, Ib, IIa) were identified in 32% of patients. The early endoscopy group had the highest rate of high-risk lesions (53%), compared to the urgent group (33%) and the late group (19%). Patients with high-risk lesions experienced significantly longer hospital stays, averaging 7.8 days compared to 4.9 days for those without high-risk findings (p < 0.005). Re-bleeding occurred in 16% of patients after initial endoscopy. Six patients received successful repeat endoscopic therapy, while two required embolization for bleeding duodenal ulcers. No further episodes of bleeding occurred in the re-bleeding group, and one patient died within 30 days of presentation, the cause was unrelated to bleeding.

**Conclusions:**

Peptic ulcer disease remains the most common cause of NVUGIB, with NSAID use as a key risk factor. Timely endoscopy and appropriate initial management are critical for optimizing outcomes. High-risk lesions are found in approximately 30% of patients and are associated with longer hospital stays. Physicians should be aware of embolization as a rescue therapy for patients who experience re-bleeding.

**Funding Agencies:**

**None**

